# Behavioral and Neuroimaging Research of Reading: a Case of Japanese

**DOI:** 10.1007/s40474-015-0066-2

**Published:** 2015-10-02

**Authors:** Taeko N. Wydell, Tadahisa Kondo

**Affiliations:** Department of Life Sciences, College of Health and Life Sciences, Brunel University London, Uxbridge, UB8 3PH UK; Faculty of Informatics, Kogakuin University, Tokyo, Japan

**Keywords:** Language universality, specificity, Reading processes, Behavioral dissociation, Neural unity, English-Japanese bilingual, Magnetoencephalography, Dyslexia

## Abstract

Behavioral studies showed that AS, an English-Japanese bilingual, was a skilled reader in Japanese but was a phonological dyslexic in English. This behavioral dissociation was accounted for by the Hypothesis of Transparency and Granularity postulated by Wydell and Butterworth. However, a neuroimaging study using magnetoencephalography (MEG) revealed that AS has the same functional deficit in the left superior temporal gyrus (STG). This paper therefore offers an answer to this intriguing discrepancy between the *behavioral dissociation* and the *neural unity* in AS by reviewing existing behavioral and neuroimaging studies in alphabetic languages such as English, Finnish, French, and Italian, and nonalphabetic languages such as Japanese and Chinese.

## Introduction

This review paper starts with a brief introduction to the Japanese orthography together with a literature review on the cognitive processes involved in reading Japanese.

### Japanese Orthography and Reading Processing

The Japanese orthography consists of two qualitatively different scripts: logographic, morphographic Kanji, derived from Chinese characters, and two forms of syllabic Kana, Hiragana and Katakana which are derived from Kanji characters [see 1–5 for more details]. These three scripts are used to write different classes of words: Kanji for nouns and root morphemes of adjectives and adverbs, Katakana for the large number of foreign load words (e.g., TV), and Hiragana for function words and the inflections of verbs, adjectives, and adverbs, and some nouns with uncommon Kanji representations.

Because of the transparent relationship between a Kana character and its pronunciation, i.e., one character represents a whole syllable/mora, it is known that children master both Kana scripts very quickly. Most children learn the Hiragana script even before they start primary school education [6–9]. Although behavioral studies showed that reading Kana involves both sequential character-by-character sub-lexical and whole-word lexical reading processes [e.g., 10, 11], Rastle et al. [11] noted that some research suggested that sub-lexical processing may be particularly strong in languages with shallower orthographies [e.g., 12, 13].

Kanji character learning is at the level of whole characters, if not at the whole word level.

Kanji learning is essentially by rote—children are introduced to new Kanji characters in texts. The learning method which is commonly in use at school is repeated writing [14] or rehearsal by writing [15] including KUSHO, literally meaning write in the air [16]. This strategy is often observed among the users of Japanese Kanji or Chinese characters [e.g., 17, 18]. By the end of compulsory education (aged between 7 and 16), a total of just above 2000 Kanji characters are taught/learnt. It should be noted, though, that adults need some 3000 characters for most everyday literacy activities [19].

Studies investigating processes involved in Kanji reading revealed both whole-word lexical and character-level sub-lexical processing taking place, although the effect size of the latter is substantially smaller than that of the former.

#### Evidence for whole-word lexical processing

Kanji word naming or semantic judgment experiments invariably showed significant word frequency/word familiarity effects, indicating the involvement of whole-word lexical processes [e.g., 2, 3, 20–23].

Shibahara, Zorzi, Hill, Wydell and Butterworth [23] also revealed a significant “imageability effect” during naming two-character Kanji words, which was indicative of whole-word level contribution in the computation of Kanji word phonology. They further reported a similar imageability effect in English (as found by Strain, Patterson, and Seidenberg [24] for English), however, the imageability effect was significantly stronger in Kanji than in English.

#### Evidence for sub-lexical processing

Patterson, Suzuki, Wydell, and Sasanuma [21] reported a case study of progressive aphasia due to Alzheimer’s disease, revealing Legitimate Alternative Reading of Component (LARC) errors in naming two-character Kanji words, whereby the pronunciation of one or more components is inappropriate for the target word but is nonetheless legitimate, and often more typical for words containing the character; this is true of many Kanji characters. Similar LARC errors in another progressive aphasic patient were reported by Fushimi, Komori, Ikeda, Patterson, Ijuin, and Tanabe [25]. These LARC errors thus indicate character-by-character sub-lexical reading processes, although neither Patterson et al. nor Fushimi et al. interpreted the data in terms of the lexical versus sub-lexical reading processing dichotomy.

In summary, reading logographic Kanji may require a greater weighting for the whole-word level contribution in the computation of phonology from orthography, as the relationship between orthography (Kanji) and phonology (pronunciation) is opaque. Learning to read in Kanji appears to be more laborious, and cognitively more demanding than that in Kana.

### Prevalence of Dyslexia

In Japan, it had been reported that the prevalence of reading difficulties or dyslexia was low:

Makita [6] first claimed through his nationwide survey that, in Japan, less than 0.1 % of children had a reading disability. Similarly, several researchers (e.g., [7, 8]) all presented evidence for the ease with which the Japanese writing system is learned. A more recent longitudinal nationwide survey across 325 primary schools in Japan conducted by Kokuritsu Tokushu-Kyouiku Sougou Kenkyujyo (Japanese National Research Institute of Special Education [26]) also revealed that less than 2 % of the children showed reading delay/impairment by the time they reached grade 6 (aged 12), the final grade in primary school education. This is higher than those found in the earlier studies, but still lower than that reported (10–12 %) in the English-speaking world [e.g., 27, 28].

More recently, Uno, Wydell, Haruhara, Kaneko, and Shinya [29] tested nearly 500 Japanese primary school children (grade 2 to grade 6) for their abilities in reading single Kana/Kanji characters/words, their vocabulary, and their other cognitive abilities, e.g., arithmetic, visual-spatial, and phonological processing skills. Their study revealed that the percentage of children with reading difficulties in the cohort differed greatly according to the type of the scripts—0.4 % for Hiragana, 1.4 % for Katakana, and 6.9 % for Kanji, respectively. Again, this is still lower that that reported in English, i.e., 10–12 %.

A similar pattern of the data was found with writing difficulties—1.6 % for Hiragana, 3.8 % for Katakana, and 6.1 % for Kanji. Significantly, in Japan, there are very few reported cases of children with reading impairments only.

Note that this study led to the development of the first standardized Screening Test of Reading and Writing for Japanese primary school children, STRAW [30].

The Japanese researchers usually attribute these reading/writing difficulties among children to “visual” or “visuospatial” processing problems rather than phonological processing problems [e.g., 31–33].

Similar findings for Chinese (another logographic orthography) are found in Wei, Bi, Chen, Liu, Weng, and Wydell [34], where the relationship between Chinese reading skills and metalinguistic awareness skills (e.g., phonological, morphological, and orthographic awareness skills) was investigated with a large cohort of normally developing preschool, grade 1, 2, and 3 children in Mainland China. Their results showed that although all three metalinguistic awareness skills significantly predicted reading success, orthographic awareness played a dominant role in the early stages of reading acquisition. Indeed, several studies argued that the major cause of developmental dyslexia in Chinese is a deficit in orthographic processing skills, rather than in phonological processing skills [e.g., 35, 36].

Thus, these results were in stark contrast with many studies in English, where phonological awareness is typically shown as the single most potent variable in literacy acquisition [e.g., 37, 38]. Ziegler and Goswami [39] further stated that many studies [e.g., 40–44] have shown that while good phonological awareness skills are the foundation for becoming good readers, poor phonological awareness skills characterize poor readers. These findings also lend support to the view that some orthographies are more prone to dyslexia, especially phonological dyslexia, than others. Some researchers [e.g., 4, 45] have argued that the discrepancy in the prevalence of dyslexia in the different languages might be primarily due to the way in which phonology is computed from orthography. In the alphabetic languages particularly in English where a finer “grain” processing of the orthography-to-phonology mapping is required (see Treiman, Mullenix, Bijeljac-Babic, and Richmond-Welty [46] for how inconsistent the orthography-to-phonology mappings are in English), developmental dyslexia forms a large minority group.

### Behavioral Dissociation

**Fig. 1:**
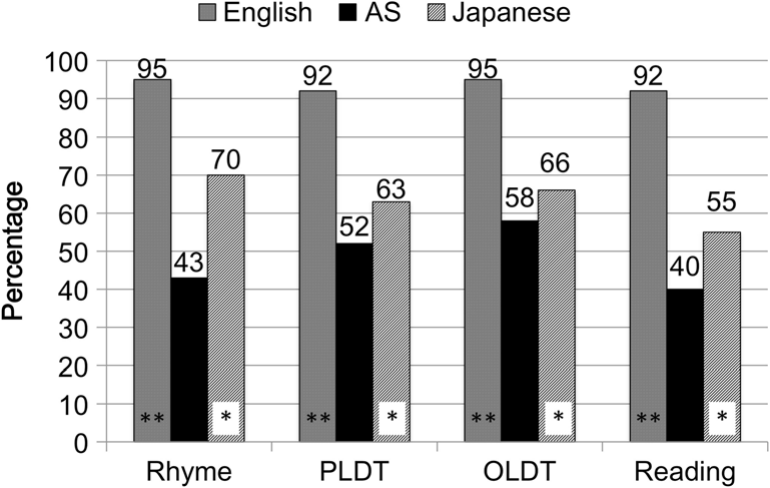
Reading/phonological tests performance by AS, and the English and Japanese control participants from Wydell and Kondo (2003). An *asterisk* denotes *p* < .05 and *two asterisks* denote *p* < .01. *Rhyme* = rhyme judgments; *PLDT* = phonological lexical decisions; *OLDT* = orthographic lexical decisions (spell check); *Reading* = reading aloud of the stimuli used in PLDT

Therefore, it is theoretically possible to see a dissociation between the reading skills in Japanese (i.e., good reading skills) and those in English (i.e., poor reading skills) in an English-Japanese bilingual. Indeed, Wydell and Butterworth [4] published a case study of such an individual, AS, whose reading skills in Japanese (Kana and Kanji) at the age of 16 were as good as those of the Japanese university students. However, his performance on reading as well as phonological tasks in English was significantly below the mean of his English and Japanese contemporaries, which is illustrated in Fig. 1. A follow-up study on AS conducted by Wydell and Kondo [5] found that his fundamental phonological deficit, which led to his phonological dyslexia in English, still persisted (see also Fig. 1) despite him successfully taking a BSc course in an English-speaking country. Wydell and her colleagues maintained that learning to read English is essentially acquiring complex mappings of sub-syllabic phonological components (i.e., phonemes) to the letter level (i.e., graphemes). Failure to acquire these sub-syllabic skills is characteristic of developmental phonological dyslexia. This is precisely what happened to AS in English.

**Fig. 2:**
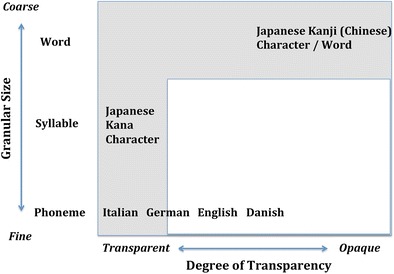
Hypothesis of Granularity and Transparency (Adapted from Wydell & Butterworth [4])

In order to account for this behavioral dissociation, Wydell and Butterworth [4] put forward the “Hypothesis of Granularity and Transparency.” The hypothesis postulates that orthographies can be described in two dimensions—“transparency” and “granularity”—with the predictions that (i) any orthography, where the print-to-sound translation is one-to-one/transparent will not produce a high incidence of phonological dyslexia regardless of the level of translation, be it phoneme, syllable, character, etc., and that (ii) even when this relationship is opaque/not one-to-one, any orthography, whose smallest orthographic unit representing sound is coarse (i.e., larger grain size) such as a whole character/whole word, will not produce a high incidence of phonological dyslexia. Therefore, any orthography used in any language can be placed in the transparency-granularity orthogonal dimension as shown in Fig. 2, and any orthography that falls into the shaded area in the figure should not produce a high incidence of phonological dyslexia.

## Neural Correlates of Reading in Different Orthographies: Language Universality (Neural Unity) vs Language Specificity (Neural Dissociation)

### Normal Readers

Paulesu, McCroy, Fazio, Menoncello, Brunswick, Cappa, Cotelli, Gossu, Corte, Lorusso, Pesnti, Gallaher, Perani, Price, D.Frith, and U.Frith [47] investigated the neural correlates of reading in different alphabetic orthographies. Under the positron emission tomography (PET) scan, English and Italian university students were asked to read words and nonwords. Their ingenious five different word and nonword stimuli in “opaque” English and “transparent” Italian consisted of (1) words either in English for English participants or in Italian for Italian participants, (2) nonwords from Italian, (3) nonwords from English, (4) international words conforming to Italian (e.g., pasta), and (5) international words conforming to English (e.g., business). They found a common distributed brain network of activation across the two languages including inferior frontal and premotor cortex, superior middle and inferior temporal gyri and fusiform gyrus on the left, and superior temporal gyrus on the right thus showing the *language universality* aspect of reading. However, Italian participants showed greater activation in left superior temporal regions, which are often implicated with *(sub-lexical)* phonological processing, while English participants showed greater activations in left posterior inferior temporal and anterior inferior frontal gyrus, which are known to be associated with word retrieval *(whole-word lexical processing)* during reading thus showing the *language specificity aspect of reading*.

Similar results to their Italian data can be found in Wydell, Vuorinen, Hellenius, and Salmelin’s [13] magnecoencephalography (MEG) study in another shallow orthography, Finnish, whereby neural correlates of length (short vs long letter strings) and lexicality (words vs nonwords) effects during reading were investigated. The results showed that the amplitude of the occipital activation at 100 ms from the onset of stimuli was significantly greater for the long letter-strings than for the short letter-strings, regardless of the lexical status of the stimuli, thus showing the length effect. Between 200 and 600 ms, the length and lexicality effects as well as the interaction between them were seen in the duration of activation (which was defined as the full width of the activation at half the maximum amplitude) in the left superior temporal lobe. Further, this sustained activation in the left superior temporal cortex persisted significantly longer for the long nonwords than other stimuli. There was no difference in the duration of the activation between the long words and short nonwords. The duration of activation was shorter for the short letter-strings than for the long letter-strings, thus showing the length effect, which was, however, significantly attenuated for the words.

It is generally agreed that letter-string length effects on naming latency reflect the sequential* (sub-lexical)* reading strategy, and the size of the effects is determined by the extent to which sub-lexical processing is involved [48]. The late (i.e., after 200 ms) length and lexicality effects in the left superior temporal lobe seen in Wydell et al. [5] could therefore result from the net neural activity of both *whole*-*word lexical* and *sub*-*lexical* reading processes. The length effect could be interpreted as on-line sequential phonological processing, which includes grapheme-to-phoneme mapping, subsequent blending, and articulatory programming. The sequential nature of the *sub*-*lexical processing* can account for the linearity of the length effects for both words and nonwords. The interpretation of the left superior temporal activation reflecting sub-lexical phonological processing is in keeping with several imaging studies of word processing (e.g., [47, 49, 50]). Like the PET study of Paulesu et al. [47], the MEG study of Wydell et al. [5] thus showed that for shallow orthographies such as Finnish and Italian at least, sub-lexical sequential reading strategy may be the optimal reading strategy and neural activation of which takes place in the left superior temporal lobe. This is different from opaque orthographies such as, for example, English.

*A language-specific aspect of neural correlates* can also be found in the functional magnetic imaging (fMRI) study conducted by Siok, Perfetti, Jin, and Tan [51] in Chinese (opaque logographic orthography). They found that the left middle frontal gyrus (LMFG) was crucial to successful Chinese reading, arguing that the LFMG functions as a “centre for fluent Chinese reading” (p.71) where typical reading processes in Chinese are mediated, i.e., the conversion of a Chinese character to a syllable, and mapping orthography (Chinese character) to semantics.

### Dyslexic Readers

Several fMRI studies in alphabetic languages such as English, French, and Italian have been conducted, whereby reading performance of normal and dyslexic readers were compared. These studies consistently showed reduced activation in left temporo-parietal areas among dyslexics when compared to normal readers (e.g., Aylward, Richards, Berninger, Nagy, Field, Grimme, Richards, Thomson, and Cramer [52]), thus showing *biological*/*neural unity*.

In contrast, the before-mentioned study of Siok et al. [51] in Chinese also revealed that when compared to Chinese normal readers, Chinese dyslexics showed reduced activation in the left middle frontal gyrus (LMFG), and instead greater activation in the left inferior prefrontal gyrus, showing *a neural dissociation* between the dyslexics in Chinese and alphabetic languages. Siok et al. [51] further argued that that “the biological abnormality of impaired reading is dependent on culture” (p. 71) and thus challenged the *biological unity theory of dyslexia* of Paulesu et al. [47]. Note that the biological unity theory of dyslexia was first advocated by Paulesu, Demonet, Fazio, McCrory, Chanoine, Brunswick, Cappa, Cossu, Habib, C.Frith, and U.Frith [53].

Moreover, as discussed earlier, it is generally accepted that, behaviorally, the successful development of Chinese reading primarily depends on orthographic processing skills [34, 36] rather than phonological processing skills, which are considered to be critical for reading alphabetic languages [e.g., 54–56]. Thus, both neuroimaging and behavioral studies in Chinese suggest a *language-specific aspect* of reading processes.

Similarly, Wang, Bi, Gao, and Wydell [57] investigated the link between dyslexia in Chinese and magnocellular functional abnormality in visual system (i.e., orthographic processing) as well as in auditory system (i.e., phonological processing). They conducted visual and auditory event-related potential (ERP) experiments using electroencephalography (EEG), eliciting mismatch negativity (MMN). The research rational was based on Stein’s [58] argument that most reading problems have a fundamental magnocellular deficit, be it auditory or visual modality. The results showed that there was no difference in the auditory MMN between the dyslexic and the control groups. However, the mean amplitude of visual MMN in the dyslexic group was significantly smaller than that of the controls, indicating that Chinese dyslexics had a deficit in the visual magnocellular pathway (i.e., orthographic skills). Thus, the ERP study of Wang et al. [57] also showed a *language-specific aspect* of neural activation.

Although, in Japanese, studies investigating neural correlates of reading in Kana and Kanji in normal readers have started to emerge [e.g., 59, 60], published neuroimaging studies on the developmental dyslexia in Japanese are currently not readily available. It will be interesting to see whether the neural correlates of reading Kanji (opaque logography), for example, would activate the left middle frontal gyrus (LMFG) as found in Chinese by Siok et al. Such neuroimaging studies in Japanese Kanji and Kana should be able to make further contribution to the scientific debates on various aspects of dyslexia in different orthographies.

### Behavioral Dissociation and Neural Unity

As discussed earlier, Wydell and Butterworth [4] and Wydell and Kondo [5] showed a clear behavioral dissociation between the ability to read in English and in Japanese Kanji/Kana in English-Japanese bilingual AS—his reading skills in English were not only poorer than the age-matched English controls but also Japanese controls, while his reading skills in Japanese were superior to his Japanese contemporaries. More recently, Wydell and Kondo (in preparation) conducted phonological lexical decision experiments using MEG with AS, and his English and Japanese controls, whereby AS and English participants were asked to respond YES to pseudohomophones such as “brane,” and similarly AS and Japanese participants were asked to respond YES to Katakana transcriptions of Kanji words (pseudohomophones in Japanese) such as “ガクセイ” for 学生/GAKU-SEI/ meaning a student. In order to respond correctly, the participants were forced to use *sequential sub*-*lexical reading strategies*.

Similar to the study of Wydell et al. [13] whereby MEG recordings were taken when Finnish participants read Finnish stimuli, the results of Wydell and Kondo showed significant neural activations in two cortical areas at two time windows across all the participants, namely, in the bilateral occipital cortex at 0–200 ms, and in the left superior temporal gyrus (STG) at 200–400 ms, respectively. No difference between AS and both the English and Japanese controls in the occipital cortices in the first time window (0–200 ms) was observed, thus indicating that AS’s dyslexia was not due to an early visual processing deficit. However, results from the later time window (200–400 ms) in the left superior temporal gyrus (STG) revealed that AS’s cortical activation was significantly weaker than that of both the English and Japanese controls, thus showing a neural unity between the two languages.

The left STG is often implicated in *sequential* (*sub*-*lexical*) *phonological processing* [e.g., 13, 47, 61, 62], phonological short-term-memory [e.g., 63], and the temporary storage of phonological information (particularly the posterior STG) [64]. Moreover, a persistent phonological short-term-memory deficit in the Landau-Kleffne’s syndrome [63], a rare acquired aphasia in children, is linked to decreased cortical activation in the STG. AS’s behavioral data also revealed a phonological short-term-memory deficit [4] as with the other studies of phonological dyslexia [28].

Finally, we need to account for the discrepancy between AS’s behavioral data showing a *behavioral dissociation* between his poor reading skills in English and his superior reading skills in Japanese, and his MEG data showing a *neural unity* between reading in English and Japanese—both showing similarly weak cortical activation in the left STG. This is because the cognitive processing demand for *transparent* syllabic Kana (larger grain size) is less than that for quasi-regular *opaque* English (smaller grain size) [28, 39, 45]. The latter requires a finer processing capability, e.g., segmenting the letter-strings into graphemes, converting these graphemes into phonemes, and finally blending the phonemes into a word or a nonword. Therefore, reading Kana may not require the neural resources in the left STG as much as reading English might do, and therefore the same functional deficit in the left STG in AS does not necessarily cause the same behavioral deficit seen in English, when AS reads in Japanese.
